# Calcium Alginate and Calcium Alginate-Chitosan Beads Containing Celecoxib Solubilized in a Self-Emulsifying Phase

**DOI:** 10.1155/2016/5062706

**Published:** 2016-04-03

**Authors:** Lorena Segale, Lorella Giovannelli, Paolo Mannina, Franco Pattarino

**Affiliations:** Dipartimento di Scienze del Farmaco, Università del Piemonte Orientale, Largo Donegani 2, 28100 Novara, Italy

## Abstract

In this work alginate and alginate-chitosan beads containing celecoxib solubilized into a self-emulsifying phase were developed in order to obtain a drug delivery system for oral administration, able to delay the drug release in acidic environment and to promote it in the intestinal compartment. The rationale of this work was linked to the desire to improve celecoxib therapeutic effectiveness reducing its gastric adverse effects and to favor its use in the prophylaxis of colon cancer and as adjuvant in the therapy of familial polyposis. The systems were prepared by ionotropic gelation using needles with different diameters (400 and 600 *μ*m). Morphology, particle size, swelling behavior, and* in vitro* drug release performance of the beads in aqueous media with different pH were investigated. The experimental results demonstrated that the presence of chitosan in the formulation caused an increase of the mechanical resistance of the bead structure and, as a consequence, a limitation of the bead swelling ability and a decrease of the drug release rate at neutral pH. Alginate-chitosan beads could be a good tool to guarantee a celecoxib colon delivery.

## 1. Introduction

Drug delivery systems containing biodegradable natural polymers are the object of more and more research studies considering the advantages that these materials can offer [1]. Among them, those containing alginate and chitosan have been widely exploited in pharmaceutical field [2–4].

Alginate is a water soluble natural biopolymer extracted from brown algae and composed of alternating blocks of 1-4 *α*-L-guluronic and *β*-D-mannuronic acid residues [5]. This polymer forms hydrogels in presence of divalent cations like Ca^2+^, Ba^2+^, Sr^2+^, and Zn^2+^ [6, 7] and this characteristic allows preparing drug loaded beads [8]. The mechanism of this gelation process involves guluronic residues with the specific chelation of Ca^2+^ forming the so-called “*egg-box*” structure [9]. Many researchers focused their attention on the development of calcium alginate beads as controlled drug delivery systems for the oral administration of drug molecules and proteins [10–12].

Chitosan is a biocompatible, biodegradable, nontoxic, linear polysaccharide composed of D-glucosamine and N-acetyl-D-glucosamine units linked by *β*-(1-4) glycosidic linkages [13]. Chitosan can be derived by partial deacetylation of chitin from crustacean shells and it is widely used for cell culture, drug delivery, and food additives [14–16].

Cross-linking of alginate and chitosan in a hydrogel is used to provide materials useful for medical and pharmaceutical applications; the obtained systems are characterized by enhanced stability compared to those obtained with a single polymer [17, 18]. In controlled drug delivery alginate-chitosan polyelectrolyte complex has received much attention in recent years [12, 19–22]. The two polymers form the polyelectrolyte complex via the ionic interaction between the carboxyl residues of alginate and the amino residues of chitosan. The alginate-chitosan beads can be produced by different methods: the two-step procedure and the one-step procedure [23]. In the first one, calcium alginate gel beads are produced by dropping a solution of alginate into a gelling bath containing calcium ions. The resulting beads are then transferred into a chitosan solution to form the membrane on their surface. The one-step procedure requires that the droplets of alginate solution fall into an aqueous solution containing both the gelling agent for alginate (e.g., calcium ions) and chitosan [24]. The choice of the production method is responsible for the properties of the beads because of the amount of bound chitosan in the resulting product. At the same time, the characteristics of the beads are affected by the molecular weight of the selected polymers and/or by the percentages of the various residues in the polymeric molecules [25, 26].

Celecoxib, a fluorinated benzenesulfonamide derivative, is a nonsteroidal anti-inflammatory drug (NSAID) with a highly selective cyclooxygenase-2 (COX-2) inhibitory action. It possesses anti-inflammatory, analgesic, and antipyretic activities due to the inhibition of prostaglandin synthesis catalyzed by COX-2. Recently, this drug was frequently investigated for its anticancer activity using* in vitro* and* in vivo* models [27–30]. Preclinical studies on celecoxib reported prominent anticancer activity against head and neck squamous cell carcinoma, colon cancer, breast cancer, and lung cancer [27, 28].

In this work alginate and alginate-chitosan beads containing celecoxib solubilized into a self-emulsifying phase were developed in order to obtain a drug delivery system for oral administration, able to delay the drug release in acidic environment and to promote it in the intestinal compartment. The rationale of this work was linked to the desire to improve celecoxib therapeutic effectiveness reducing its gastric adverse effects and to favor its use in the prophylaxis of colon cancer and as adjuvant in the therapy of familial polyposis [31–33]. The aim of the current study was the evaluation and the comparison of the properties of celecoxib loaded calcium alginate and calcium alginate-chitosan beads. Morphology, particle size, swelling behavior, and* in vitro* drug release performance of beads in aqueous media with different pH were investigated.

## 2. Materials and Methods

### 2.1. Materials

Celecoxib was obtained from Chemos GmbH (Regenstauf, Germany). Anhydrous calcium chloride and sodium alginate (molecular weight 120000–190000 g/mol; 1.56 mannuronic-guluronic residues ratio) were purchased from Sigma Aldrich (Milan, Italy), while low molecular weight chitosan was from Fluka (Milan, Italy). Labrasol (caprylocaproyl macrogol-8 glycerides) was a gift of Gattefossè (Milan, Italy); TPGS (D-*α*-tocopheryl polyethylenglycol 1000 succinate) was kindly donated by Isochem (Gennevillers, France). All other chemicals were of analytical grade.

### 2.2. Preparation of Calcium Alginate Beads

Calcium alginate beads were prepared by gelation method using calcium ions as cross-linking agent. In detail, a 1.5% (w/w) sodium alginate aqueous solution was mixed with a drug loaded self-emulsifying phase in 4 : 1 ratio and added drop by drop to a 100 mM CaCl_2_ solution [34]. The self-emulsifying phase was prepared mixing weighed amounts of Labrasol and TPGS at 50°C and dissolving celecoxib in the excipient solution. The emulsion (sodium alginate solution and self-emulsifying phase) was manually extruded in the hardening bath through needles with 400 or 600 *μ*m inner diameter, under constant gentle stirring, at room temperature. After 15 minutes, the beads were collected, washed with deionised water to eliminate the excess of calcium ions and then dried at 40°C overnight. The composition of prepared formulations, coded CAl 600 and CAl 400, was listed in Table 1.

### 2.3. Preparation of Calcium Alginate-Chitosan Beads

Calcium alginate-chitosan beads (identified as CAlCh 600 and CAlCh 400) were prepared according to the one step method. The procedure was identical to that adopted in the case of alginate beads with the exception that the hardening bath was a 0.2% (w/w) chitosan solution in diluted acetic acid (1%) containing CaCl_2_ at a concentration of 100 mM. The composition of the chitosan formulations was reported in Table 1.

### 2.4. Morphological and Particle Size Analysis

The morphology of the wet and dried beads and the particle size of the dried beads were analysed using a Motic SMZ168 stereomicroscope and an image analysis software (Motic Image Plus 2.0). For each formulation, the particle size was calculated as the average value of the size of 20 dried particles.

### 2.5. Drug Content

Six milligrams of drug loaded dried beads was solubilized in phosphate buffer solution (100 mL) at pH 6.8 added of 0.75% (w/v) sodium laurylsulphate at 70°C under stirring for two hours. After cooling, the obtained solutions were filtrated and analysed spectrophotometrically at 255 nm; the results are the average of at least three determinations.

### 2.6. Swelling Study

Swelling studies were carried out at 37°C on dried beads put into three aqueous media characterized by different pH: hydrochloric acid at pH 1.0 and phosphate buffer at pH 6.8 and pH 7.4.

Accurately weighed amounts of calcium alginate and calcium alginate-chitosan dried beads were put in glass vials containing 5 mL of each fluid. After fixed time intervals (5, 15, 30, 60, and 120 minutes), the samples were recovered, gently wiped with paper, and weighed again. The dynamic weight change of the beads with respect to time, defined as swelling degree (Sw), was calculated according to the following equation:

(1) $$\begin{array}{c} Sw\%=\frac{{W_{t}-W}_{0}}{W_{0}}\times 100, \end{array}$$

where *W*_*t*_ is the weight of the beads in the swollen state at time *t* and *W*_0_ is the initial weight of the dried beads [35].

### 2.7. Celecoxib Release Study

The* in vitro* release studies were performed in hydrochloric acid at pH 1.0 and in phosphate buffer at pH 6.8 and at pH 7.4 added of 0.75% sodium laurylsulphate to guarantee the maintenance of the sink conditions. The studies were carried out by placing accurately weighed amounts of each formulation, equivalent to 8 mg of celecoxib, in 500 mL of the selected fluid at 37°C under a rotation rate of 100 rpm (apparatus 2, paddle). Filtered samples were withdrawn at specific time intervals without replacement and analysed for celecoxib content using an UV spectrophotometer at 255 nm when the fluid was HCl and phosphate buffer at pH 6.8 or at 256 nm in the case of phosphate buffer at pH 7.4. Each experiment was done in triplicate.

The drug release performances of calcium alginate and calcium alginate-chitosan beads were compared using the dissolution parameters* t*10%,* t*50%, and* t*90% which indicate the time points at which 10%, 50%, and 90% of the drug were released [36] and the* f2* similarity parameter [37]. For curves to be considered similar* f2* values should be close to 100, and* f2 *values greater than 50 (50–100) ensure sameness or equivalence of the two curves.

### 2.8. Statistical Analysis

The results were statistically analysed to test significant differences by Student's* t*-test, at 95% confidence interval; *p* values less than 0.05 were considered statistically significant.

## 3. Results and Discussion

Alginate and alginate-chitosan beads were obtained by ionotropic gelation method dropping an emulsion, composed of alginate aqueous solution and the drug loading self-emulsifying phase, through 23 G (600 *μ*m) or 27 G (400 *μ*m) needles, in a calcium chloride or in a calcium chloride-chitosan gelling bath. The excipients selected for the self-emulsifying phase were Labrasol, a liquid component with self-emulsifying and solubility enhancer properties, and D-*α*-tocopheryl polyethylenglycol 1000 succinate as coemulsifying and absorption enhancer agent (Table 1). The composition of the self-emulsifying phase was the same used in a previous work [34].

**Figure 1:**
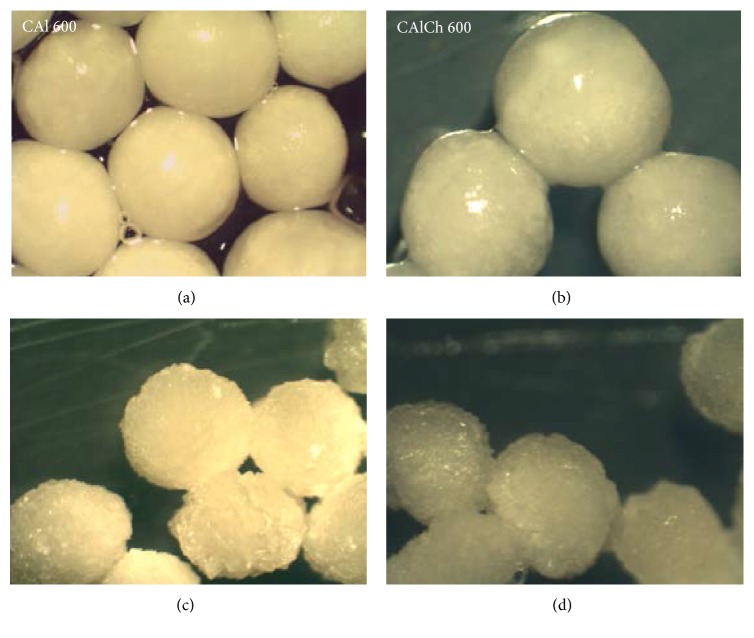
Stereomicroscopic images of wet (a-b) (2x magnification) and dry (c-d) (3x magnification) beads obtained using a needle of 600 *μ*m in diameter.

**Figure 2:**
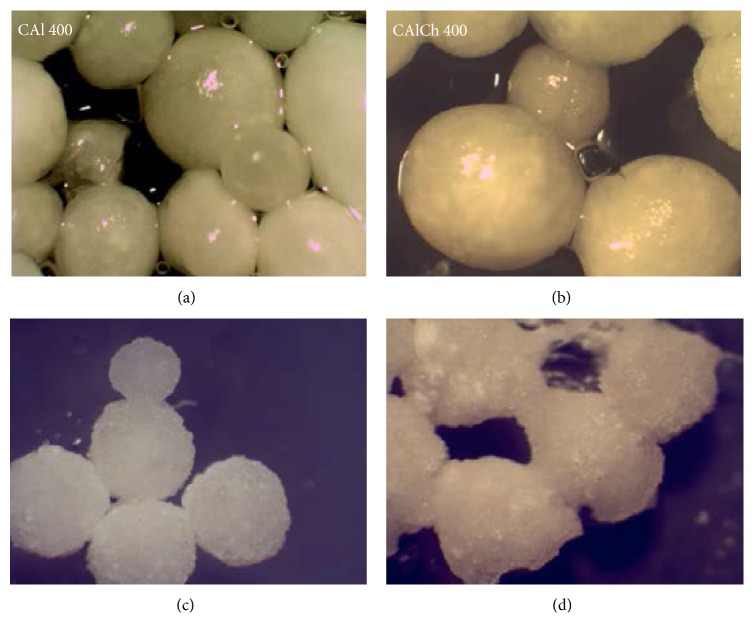
Stereomicroscopic images of wet (a-b) (2x magnification) and dry (c-d) (3x magnification) beads obtained using a needle of 400 *μ*m diameter.

Stereomicroscopic images of wet and dry alginate and alginate-chitosan beads are reported in Figures 1 and 2. Immediately after the preparation CAl 600 beads (Figure 1(a)) show regular shape and homogeneous dimensions; they are white and opaque with a smooth, glossy, and homogeneous surface.

The drying process does not change the shape of beads but leads to the reduction of their dimensions and modifies the characteristics of their surface, which is irregular and wrinkled (Figure 1(b)). The loss of water induces a decrease of the distance between the polymeric chains and a variation of the structure of the beads, which is not compact and continuous but composed of small micronuclei adhering to each other. The fully swollen CAlCh 600 particles are slightly yellow and quite regular in shape and show a smooth surface (Figure 1(c)). In this case, the drying process affects the spherical shape of the beads (Figure 1(d)); they become ellipsoidal; their dimension decreases and their surface is very rough. Moreover, after drying a partial surface agglomeration of the beads is observed: it is attributable to the adhesive properties of chitosan [38]. In the case of beads prepared using a 400 *μ*m needle (Figures 2(a)–2(c)), the images recorded immediately after the preparation give evidence that for both the formulations (with or without chitosan) particles are not homogeneous in diameters even if they are regular in shape. CAl 400 and CAlCh 400 dry beads (Figure 2(b)) have a surface characterized by asperity and concavity; moreover, in the case of alginate-chitosan formulation the particle shape is completely irregular and the presence of solid bridges of chitosan is well evident which bind the beads impeding their separation.

Average size of celecoxib loaded beads is between 715 and 896 *μ*m (Table 2). The diameter of the beads was significantly affected by the diameter of the needle used during preparation (*p* < 0.05). The addition of chitosan to alginate beads changes significantly their dimensions only when the 400 *μ*m needle was used (*p* < 0.05). Moreover, as indicated by the high values of the standard deviation, using the 400 *μ*m needle the final product is a family of particles inhomogeneous in dimensions.

All the formulations contain high amount of drug (Table 2) homogeneously distributed in the excipient matrix and the differences among their drug content were not significant except for CAlCh 400 slightly lower than the others (*p* < 0.001). The percentage of celecoxib in the beads exceeds the theoretical value and this is due to the loss of Labrasol during the curing time [34], justifiable considering the high affinity between this excipient and water which drives it out of the beads into the gelling bath.

**Figure 3:**
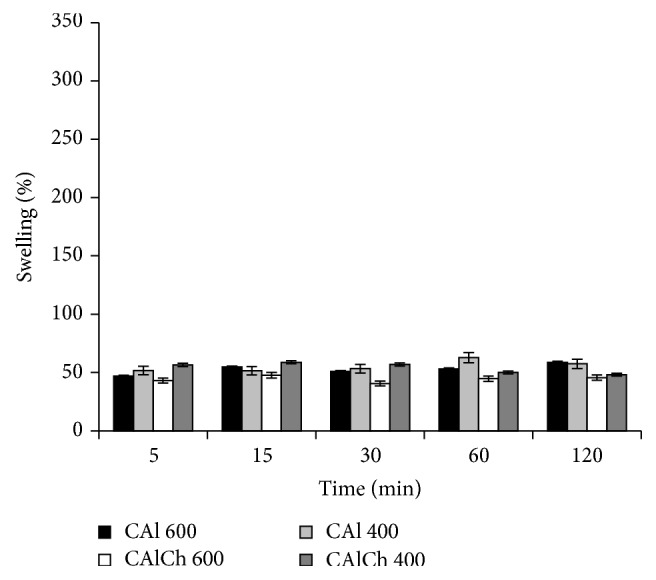
Swelling degree in hydrochloric acid at pH 1.0.

**Figure 4:**
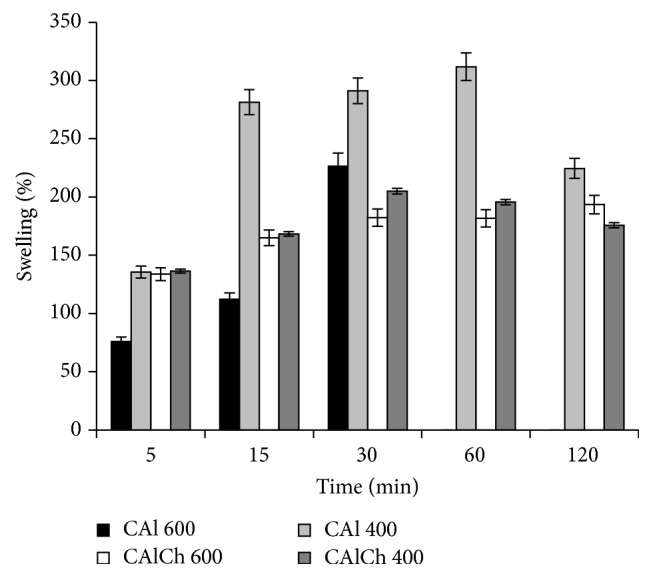
Swelling degree in phosphate buffer at pH 6.8.

**Figure 5:**
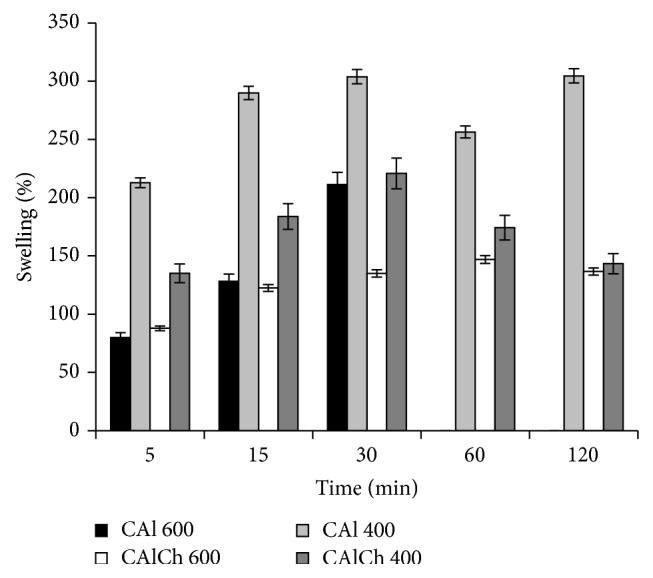
Swelling degree in phosphate buffer at pH 7.4.

A peculiar property of alginate or alginate-chitosan microparticulate systems in the dry form is their ability, after contact with an aqueous fluid, to rehydrate, take up the fluid, and undergo a swelling process, mainly associated with the hydration of the hydrophilic groups of polymers. When the fluid is water, it penetrates into the particles filling the pores among the polymeric chains and causing an important swelling of the system, without erosion/disintegration. Selecting fluids with different pH, the swelling behavior of the beads could change. For this reason, the swelling ability of calcium alginate and of calcium alginate-chitosan beads has been evaluated in HCl at pH 1.0 and in phosphate buffer solutions at pH 6.8 and 7.4 (Figures 3–5).

In acidic environment (Figure 3), there are no differences in the swelling ability of the four formulations; at this pH, the maximum swelling degree is not over 60%. The alginate and alginate-chitosan beads absorb part of the fluid; their weight initially increases and then remains constant. At this pH, for the alginate systems (CAl 600 and CAl 400), the carboxylate groups of the polymer localized on the surface of particles are protonated and a layer of alginic acid forms. The insolubility of alginic acid in this fluid and the formation of hydrogen bonds, responsible for an increase of the structure stability, impede the penetration of additional fluid into the deeper layers of particles, limiting their swelling. The same swelling behavior is observed for CAlCh 600 and CAlCh 400 systems. Even if, in acidic environment, chitosan is highly soluble and charged for the conversion of its amine units into NH^3+^ soluble form, the interaction of amino groups and protonated carboxylic groups is not strong enough to promote swelling. Thus, the limited total swelling behavior is dominated by calcium alginate structure.

Figures 4 and 5 show that the formulations exhibit high swelling ability at pH 6.8 and pH 7.4. For CAl 600 formulation, the weight of particles grows rapidly, reaches a peak after 30 minutes, and then decreases abruptly because of the erosion/disintegration of the system. This behavior can be due to ion exchange reaction between Na^+^ (present in the phosphate buffer) and Ca^2+^ linked to carboxylic groups of alginate. Monovalent ions replace bivalent ones causing the breakup of the “egg-box” structure and the increase of the distance between the polymeric chains and favoring the fluid absorption and the swelling of the systems. This process goes on until the osmotic pressure into the beads balances the strength of the cross-linking bonds and physical entanglements, which preserve the structure of the beads. Thus, particles start to disintegrate and their weight diminishes.

The results obtained from the swelling study evidence that CAlCh 600 and CAlCh 400 beads are characterized by a more resistant structure compared to CAl 600 and CAl 400, probably attributable to the interactions between alginate and chitosan chains. The maximum swelling degree of chitosan beads is lower than that of the alginate; alginate-chitosan systems are able to reach a swelling equilibrium in about 30 minutes and to maintain their weight at a constant level until the end of the test. Probably the interactions between the two polymers are responsible for the formation of particles with a considerable mechanical resistance, which limits the fluid uptake and the structure disintegration. Finally, comparing the swelling behavior of CAl 400 versus CAl 600 and CAlCh 400 versus CAlCh 600 beads (same composition, different diameter of the needle used in the preparation process), it is possible to note that in phosphate buffers CAl 400 and CAlCh 400 reached a swelling maximum peak higher than that of CAl 600 and CAlCh 600, respectively.

**Figure 6:**
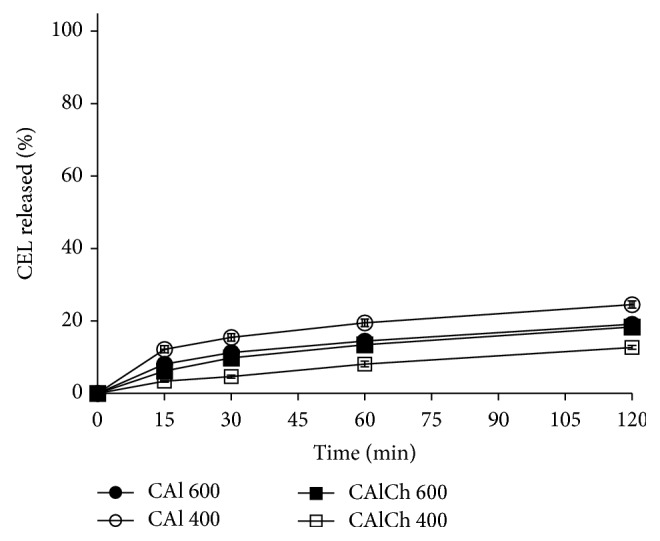
Celecoxib release profiles in hydrochloric acid at pH 1.0.

**Figure 7:**
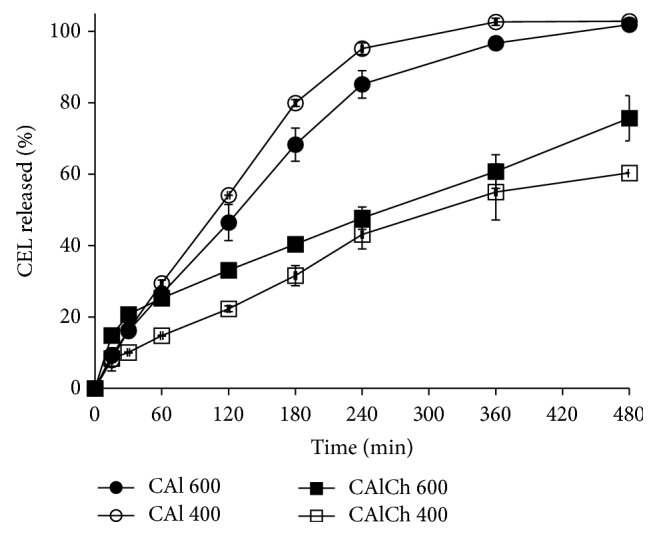
Celecoxib release profiles in phosphate buffer at pH 6.8.

**Figure 8:**
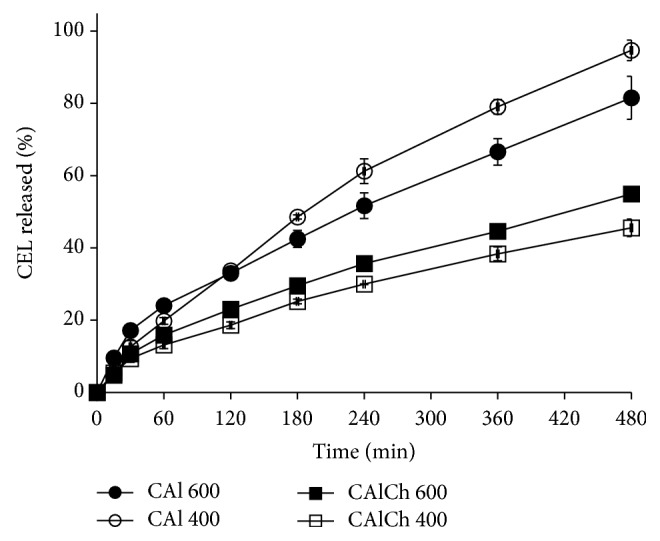
Celecoxib release profiles in phosphate buffer at pH 7.4.

The drug release profiles obtained from the different formulations at pH of 1.0, 6.8, and 7.4 are shown in Figures 6–8. The* in vitro* celecoxib release is affected by the pH of the selected fluid: the percentage of drug released in acidic medium in two hours is quite low and varies between 12.70% and 24.53% (Figure 6). The delay of celecoxib release can be ascribed to the reduced swelling ability of the systems in this fluid; neither the composition of the beads nor their diameter affects the drug release performance (*f2* parameter values always over 50). At pH 1.0, the release process is governed only by the diffusion of the drug. This result allows satisfying the first object of this research work that is to minimize drug delivery in acidic environment to promote and favor this process at intestinal level.

In phosphate buffer at pH 6.8, the formulations are characterized by a drug release behavior affected by their composition and not significantly by their dimensions (Figure 7). In this fluid, the systems initially swell and then erode/disintegrate and, consequently, the drug release process is driven at first by diffusion and then by the polymeric relaxation. Alginate beads (CAl 600 and CAl 400) are able to complete the celecoxib release in about eight hours; on the contrary no more than 75% of the drug loaded in alginate-chitosan beads passes in solution after the same time. The comparison of the results from alginate and alginate-chitosan microparticle formulations reveals that the profiles are not similar having* f2 *values lower than 50. A possible explanation for such a behavior is the electrostatic interaction between carboxyl groups of the alginate and the amino group of chitosan that improves the mechanical resistance of the polymeric network reducing its swelling and erosion at pH 6.8.

Surprisingly, the celecoxib release rate slows down mainly for CAlCh 400 formulation even if this formulation is characterized by the lowest particle size. Probably the small diameter and the high surface area of these particles lead to the formation of a thicker chitosan layer around the beads, which opposes a great resistance to the fluid uptake and, as a consequence, to the drug release.

Also at pH 7.4 the differences in the drug release performances can be attributed to the formulation composition rather than to the particle dimensions (Figure 8). All the formulations showed an almost constant drug release rate. There are no differences between CAl 600 and CAl 400 and between CAlCh 600 and CAlCh 400 release curves; when chitosan is in the beads, the drug release rate decreases.

The same conclusions are shown by the analysis of the results of the drug release through the dissolution parameters (*t*10%,* t*50%, and* t*90%) (Table 3). The differences in the release behavior of the beads are detected also through the dissolution parameters* t*10%,* t*50%, and* t*90% and are well evident for the time necessary to release 50 and 90% of the loaded drug. In phosphate buffer solutions, alginate-chitosan beads require longer times to deliver 50 and 90% of celecoxib compared to alginate beads. Probably the presence of the alginate-chitosan complex provokes a growth of the structural wrinkles, irregularity and complexity of the bead structure, which make the drug liberation difficult.

## 4. Conclusions

The investigated celecoxib loaded alginate and alginate-chitosan beads minimize the drug release in acidic environment favoring this process at intestinal pH (6.8 and 7.4). The experimental results demonstrate that the presence of chitosan in the formulation is responsible for an increase of the resistance of the bead structure and, as a consequence, for a limitation of the bead swelling ability and for a decrease of the drug release rate at neutral pH. Alginate-chitosan beads could be a valuable vehicle of celecoxib for dosage forms useful as adjuvant therapy in patients with familial polyposis and precancerous disease of colon.

## Figures and Tables

**Table 1:** Composition of calcium alginate and calcium alginate-chitosan beads.

Formulation	Sodium alginate (% w/w)	Self-emulsifying phase (% w/w)	Gelling bath	Needle diameter (*μ*m)
Cal 600	1.5	Celecoxib 27.4	CaCl_2_ 100 mM	600
		Labrasol 68.5		
		TPGS 4.1		

Cal 400	1.5	Celecoxib 27.4	CaCl_2_ 100 mM	400
		Labrasol 68.5		
		TPGS 4.1		

CAlCh 600	1.5	Celecoxib 27.4	CaCl_2_ 100 mM + 0.2% chitosan	600
		Labrasol 68.5		
		TPGS 4.1		

CAlCh 400	1.5	Celecoxib 27.4	CaCl_2_ 100 mM + 0.2% chitosan	400
		Labrasol 68.5		
		TPGS 4.1		

**Table 2:** Average diameter and celecoxib content of the dry beads.

	Diameter (*μ*m)	Drug content (%)
CAl 600	896 ± 64.24	42.10 ± 1.30
CAl 400	715 ± 80.96	43.63 ± 0.77
CAlCh 600	881 ± 66.87	40.94 ± 1.37
CAlCh 400	795 ± 103.70	39.78 ± 0.66

**Table 3:** Time (min) necessary to release 10, 50, and 90% of the loaded drug.

	CAl 600	CAl 400	CAlCh 600	CAlCh 400
HCl				
*t*10%	24	12	31	85
*t*50%	—	—	—	—
*t*90%	—	—	—	—

pH 6.8				
*t*10%	16	19	10	30
*t*50%	130	110	261	310
*t*90%	290	220	>8 h	>8 h

pH 7.4				
*t*10%	16	25	28	35
*t*50%	229	187	423	>8 h
*t*90%	>8 h	444	>8 h	>8 h
